# A cuproptosis-related lncRNA signature to predict prognosis and immune microenvironment of colon adenocarcinoma

**DOI:** 10.1038/s41598-023-33557-6

**Published:** 2023-04-18

**Authors:** Dongming Li, Guangzhen Qu, Shen Ling, Yuanlin Sun, Yingnan Cui, Yingchi Yang, Xueyuan Cao

**Affiliations:** 1 Department of Gastric and Colorectal Surgery, General Surgery Center, The First Hospital of Jilin University, Changchun, 130021 Jilin China; 2 Department of Interventional Radiology, Beijing Chaoyang Hospital, Capital Medical University, Beijing, 100020 China; 3 Department of General Surgery, Beijing Friendship Hospital, Capital Medical University, National Clinical Research Center for Digestive Diseases, Beijing, 100050 China

**Keywords:** Computational biology and bioinformatics, Computational models, Cancer genetics, Gastrointestinal cancer, Tumour biomarkers

## Abstract

Cuproptosis is a novel cell death modality but its regulatory role in the colon cancer remains obscure. This study is committed to establishing a cuproptosis-related lncRNA (CRL) signature to forecast the prognosis for colon adenocarcinoma (COAD). The Cancer Genome Atlas (TCGA) samples were randomly divided into training and validation cohorts. LASSO-COX analysis was performed to construct a prognostic signature consisting of five CRLs (AC015712.2, ZEB1-AS1, SNHG26, AP001619.1, and ZKSCAN2-DT). We found the patients with high-risk scores suffered from poor prognosis in training cohort (*p* < 0.001) and validation cohort (*p* = 0.004). Nomogram was created based on the 5-CRL signature. Calibration curves, receiver operating characteristic (ROC) curves, and decision curve analysis (DCA) demonstrated the nomogram performed well in 1‑, 3‑, and 5‑year overall survival (OS). Subsequently, we observed increased infiltration of multiple immune cells and upregulated expression of immune checkpoints and RNA methylation modification genes in high-risk patients. Additionally, gene set enrichment analysis (GSEA) revealed two tumor-related pathways, including MAPK and Wnt signaling pathways. Finally, we found AKT inhibitors, all-trans retinoic acid (ATRA), camptothecin, and thapsigargin had more sensitivity to antitumor therapy in high-risk patients. Collectively, this CRL signature is promising for the prognostic prediction and precise therapy of COAD.

## Introduction

Evidence from cancer statistics clearly states that colorectal cancer (CRC) has the third highest incidence and second highest mortality among many infamous tumors^1^. Meanwhile, CRC incidence and morbidity are still increasing rapidly, especially in many low-income and middle-income countries^2,3^. Despite considerable advances in screening, diagnosis, surgical techniques, and comprehensive pharmacological interventions, the 5-year relative survival rate for CRC has improved by only 14% over the past few decades^4^. Colon adenocarcinoma (COAD) is one of the most important pathological types of CRC, with occurring of distant metastasis in approximately 20% of patients who face the challenge of difficult treatment and poor prognosis^5^. Thus, we still need to explore new methods to improve and predict the prognosis of COAD.

Recently years, apoptosis, autophagy, pyroptosis, ferroptosis, and other cell death pathways have been discovered successively^6^. Different from the forementioned types of cell death, recent studies disclosed a novel copper-induced modality of mitochondrial cell death, which was defined as cuproptosis^7,8^. Copper is classified as a metallic trace element that is indispensable for all organisms. Abundant observations decipher that the copper levels both in serum and cancerous tissues of CRC patients are significantly increased compared to healthy counterparts^9–11^. These copper plays important regulatory roles in several essential processes of tumor progression, including cell proliferation, angiogenesis, and metastasis^12–14^. Nevertheless, too much copper in cells can trigger copper-related cell death^15^. When excessive intracellular copper accumulates, it will cause the polymerization of mitochondrial lipoylated proteins and the subsequent loss of iron-sulfur cluster proteins in mitochondria, which will trigger cell death eventually^7^.

With the concept of cuproptosis proposed, an increasing number of researchers are committed to exploring its relevance to tumor. According to previous view, increasing the concentration of copper in tumor cells produces its tumoricidal effect through triggering ROS-mediated oxidative stress pathways^16^. However, subsequent findings revealed the known inhibitors of other cell death modalities could not reverse cuproptosis of tumor cells^7,17^. Consequently, copper-induced cell death is considered to be an independent form of cell death. The latest view is that elevated copper can directly bind to lipoylated dihydrolipoamide *S*-acetyltransferase (DLAT), an enzyme participating in the formation of the pyruvate dehydrogenase complex and affecting the mitochondrial tricarboxylic acid (TCA) cycle, which results in the oligomerization of lipoylated DLAT, subsequently leading to proteotoxic stress and tumor cell death^18–20^. Additionally, p53, a tumor suppressor protein, might increase sensitization to cuproptosis by enhancing mitochondrial metabolism and inhibiting glycolysis pathways^21^. Notably, in vitro and in vivo experiments have confirmed the inhibition of aerobic glycolysis by targeting glyceraldehyde 3-phosphate dehydrogenase promotes cuproptosis of CRC^22^. Nevertheless, since the real mechanism of cuproptosis has just been revealed, the detailed roles and potential signaling pathways of cuproptosis on cancer still needs further study.

The researchers defined non-coding RNAs longer than 200 nucleotides as long non-coding RNAs (lncRNAs) that participate in the development and progression of tumors^23^. Evidence indicates that lncRNAs act a pivotal part in regulating the cell cycle, proliferation, apoptosis, epithelial-mesenchymal transition, metastasis, and drug susceptibility in COAD^24^. Therapies targeted lncRNA in tumor microenvironment (TME) are expected to provide novel strategies for cancer treatment.

Currently, multiple lncRNAs are identified as prognostic indicators of colon cancer, involved in necroptosis^25^, ferroptosis^26^, and pyroptosis^27^. However, the regulatory roles of cuproptosis-related lncRNAs (CRLs) for COAD remain obscure. In this work, we established a CRL signature to forecast the survival of COAD utilizing the TCGA database. Additionally, we comprehensively analyzed the association between CRLs and tumor microenvironment, RNA methylation, and drug sensitivity of COAD respectively. This CRL signature will be promising to provide clinical references for the individualized therapy and prognosis of colon cancer.

## Methods and materials

### Data acquisition

**Figure 1:**
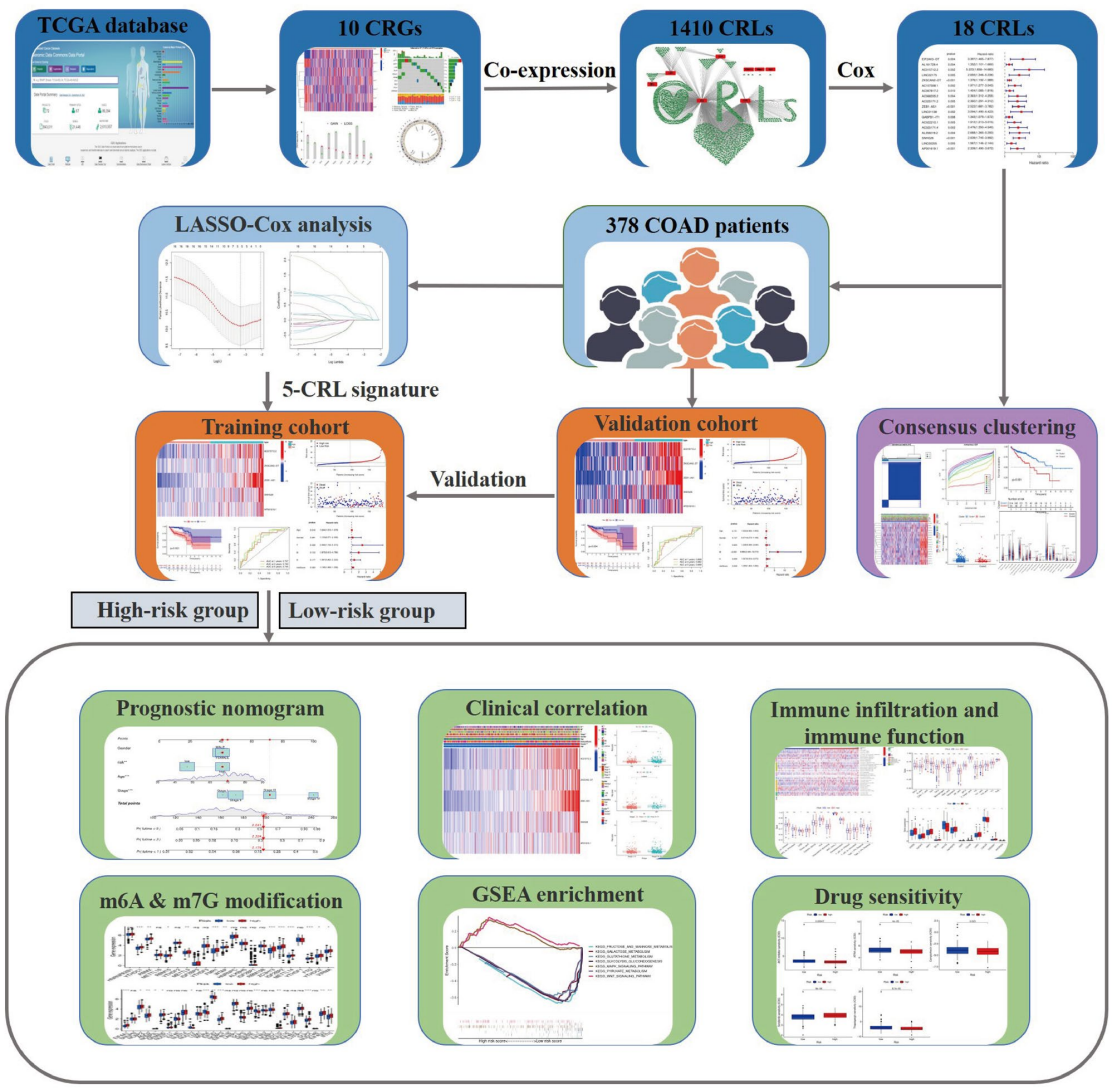
The workflow chart in this study.

We downloaded transcriptomic profiles and corresponding clinical information by The Cancer Genome Atlas (TCGA) portal (https://portal.gdc.cancer.gov/) where a total of 436 COAD samples were finally contained for this study, including 397 tumor tissues and 39 normal tissues. We also downloaded somatic mutation profiles of COAD from the TCGA data portal as well as copy number variation (CNV) profiles of COAD from UCSC Xena (https://xena.ucsc.edu/). While screening clinical characteristics, we deleted the samples with missing survival information to narrow the computational bias, and 378 COAD samples were finally included in this study. The workflow chart in this work was shown in Fig. 1.

### Expression of cuproptosis-related genes (CRGs) and identification of CRLs

There were 10 CRGs (CDKN2A, DLAT, DLD, FDX1, GLS, LIAS, LIPT1, MTF1, PDHA1, PDHB) acquired from a previous study^7^. We explored the aberrant expression of CRGs between normal and COAD samples, and investigated the landscape of CRGs alteration in the patients with COAD. Subsequently, we worked on the establishment of a co-expression network between CRGs and lncRNAs via Pearson correlation analysis with the criteria of “correlation coefficient > 0.4” and “*p* < 0.001” to filter the potential CRLs. After that, Cox regression analysis with *p* < 0.01 was put into practice to screen CRLs and estimate their impact on prognosis.

### Consensus clustering analysis of CRLs

Based on the prognostic CRLs, the “ConsensusClusterPlus” package was selected to fulfill the clustering for COAD patients and 1000 repetitions were performed to ensure the stability of the classification^28^. The correlations were explored between different clusters and clinical characteristics, including age, gender, T stage, N stage, M stage, and TNM stage. Then, the overall survival (OS) was compared between the different clusters.

### Construction and verification of cuproptosis-related prognostic signature

There were 378 COAD patients who were randomly (at a 1:1 ratio) split into training cohort (n = 190) which was utilized to build the CRL prognostic model or validation cohort (n = 188) which was utilized to check the robustness of CRL model. After the forementioned Cox regression analysis, tenfold cross-validated least absolute shrinkage and selection operator (LASSO) Cox regression analysis was utilized to further screen for CRLs with prognostic values and construct a predictive CRL signature. The signature-calculated risk scores were figured up for all COAD patients by a specialized formula: ${\mathrm{Risk score }=\Sigma }_{i=1}^{n}{\beta }_{i}\times {x}_{i}$, in which *β*_*i*_ represents the risk coefficient and *x*_*i*_ represents the corresponding expression level of CRLs.

Selecting the median risk score of the training cohort as a cutoff value, COAD samples both in the training and validation cohorts were split into high- and low-risk subgroups. Kaplan–Meier (K–M) curves and 1-, 3-, and 5-year receiver operating characteristic (ROC) curves were applied to evaluate the OS and prognostic robustness of CRL signature. To further determine the prognostic value of CRL signature, the independent risk factors were explored utilizing univariate and multivariate Cox regression analyses. Finally, we applied a nomogram to facilitate the prediction of survival for COAD patients via the “survival” and “regplot” packages. We verified the prediction accuracy of nomogram by discriminating the calibration curves and time-dependent ROC curves. To the determine clinical application prospects of the prediction model, we applied decision curve analysis (DCA) to assess the clinical net benefit of nomogram.

### Pathway enrichment analysis of CRLs

Gene set enrichment analysis (GSEA), refers to an algorithm to rank the significant differences in gene expression using a predefined gene set between two biological states, and subsequently test whether the members of the gene set are enriched at the top or bottom of the sequence^29^. The differences in Kyoto Encyclopedia of Genes and Genomes (KEGG) pathways were analyzed in different risk subgroups via the GSEA 4.2.3 software acquired from the official website (https://www.gsea-msigdb.org/gsea/index.jsp). The results of GSEA were visualized via the “ggplot2” package.

### Immune analysis of tumor microenvironment

PD-1 and PD-L1 are the most important immune checkpoints which are a strong correlation with tumor immune tolerance and immunotherapy. Based on the different clusters, we analyzed the differential expression of PD-1 and PD-L1, and reveal the differences in the distribution of 22 immune cells via CIBERSORT, an algorithm used to reflect the abundance of cellular infiltration in complicated tissues. Simultaneously, the stroma scores that quantify the distribution of stromal cells in the TME, the immune scores that quantify the distribution of immune cells in the TME, and the estimate scores that combine the distribution of stroma and immune cells, were calculated via ESTIMATE algorithm of the “estimate” package to evaluate the tumor purity and immune cell infiltration between different clusters.

Additionally, we explored the differences in the expression of immune checkpoint genes between different risk subgroups. Meanwhile, based on the signature-calculated risk scores, the differential infiltration of multiple immune cells was investigated between different risk subgroups utilizing the algorithms of CIBERSORT, CIBERSORT-ABS, QUANTISEQ, XCELL, MCPcounter, EPIC, and TIMER, respectively. Finally, to further determine whether CRL signature can change the TME of COAD, single-sample GSEA (ssGSEA) was put into practice to understand the differences in the scores of immune cells and immune function in different risk subgroups via the “GSVA” and “GSEABase” packages.

### *N*6-methyladenosine and *N*7-methylguanosine related gene analysis

*N*6-methyladenosine (m6A) and *N*7-methylguanosine (m7G) modifications are closely related to tumorigenesis and progression through affecting the expression levels of certain oncogenes and antioncogenes^30,31^. To understand the correlation between RNA methylation and CRLs signature, we analyzed differences in m6A- and m7G-related gene expression in different risk subgroups and visualized the correlation via the “ggplot2” package.

### Drug susceptibility prediction

To determine the drug susceptibility of chemotherapy and targeted therapy in different risk subgroups, the half inhibitory concentration (IC50) of a series of therapeutic drugs was contradistinguished based on the “pRRophetic” package.

### Statistical analysis

The establishment of a co-expression network between CRGs and lncRNAs via Pearson correlation analysis with the criteria of “correlation coefficient > 0.4” and “*p* < 0.001”. Cox and LASSO-Cox regression analysis were utilized to establish the prognostic CRL signature. K–M curves and log-rank test were manipulated to evaluate the OS in different groups. ROC curves were manipulated to determine the predictive robustness of CRL signature. Basic clinical characteristics of COAD patients between the training and validation cohorts were compared utilizing the Chi-square test. R 4.2.0 software performed all statistical analysis. Two-tailed *p* < 0.05 was considered statistically significant.

## Results

### Expression and mutation of cuproptosis-related genes in COAD

**Figure 2:**
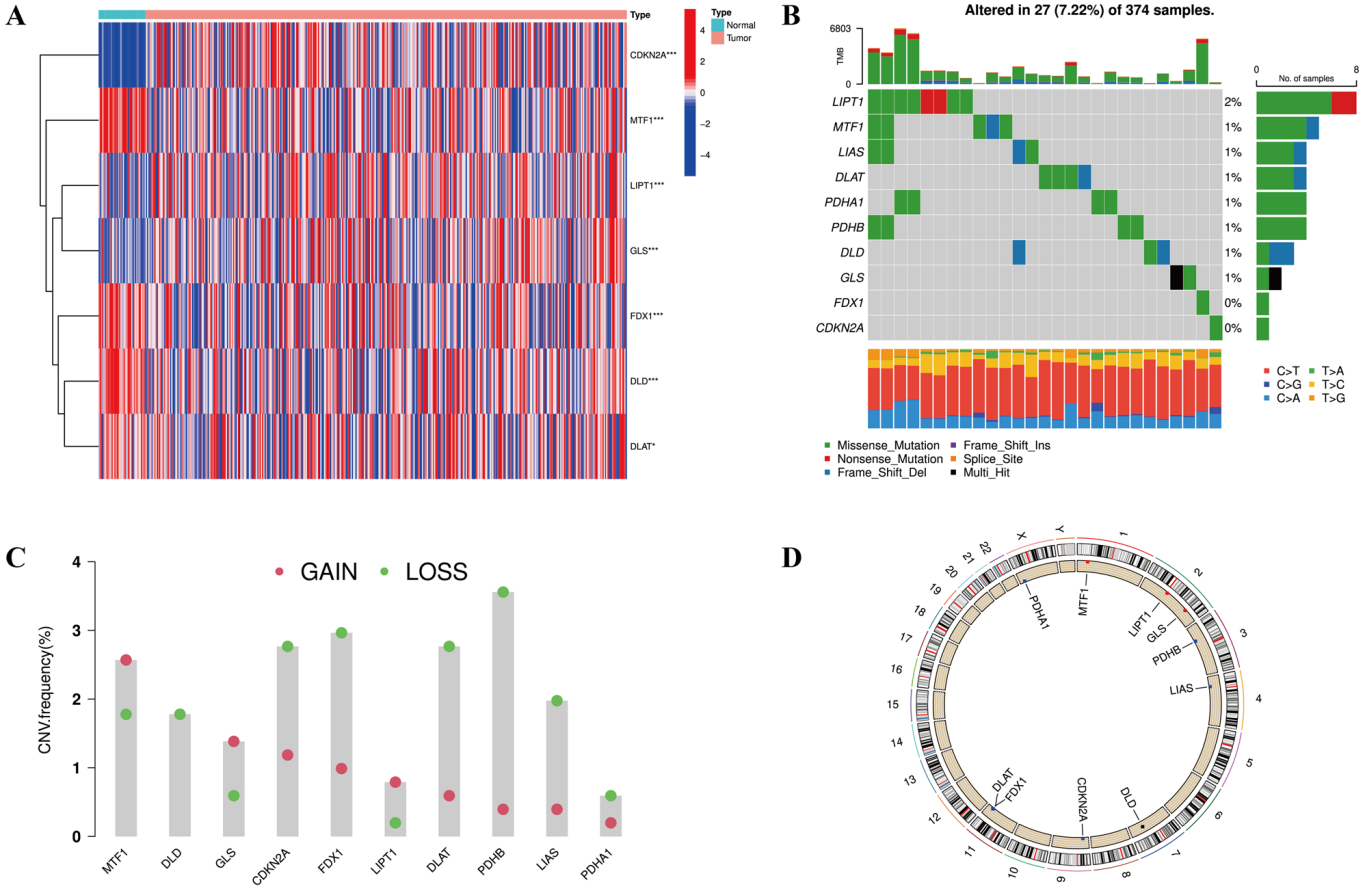
Cuproptosis-related gene (CRG) expression and mutations in COAD samples. (**A**) The differences in CRG expression between normal and tumor tissues. *p < 0.05, and ***p < 0.001. (**B**) The landscape of CRG mutations in 374 COAD samples. (**C**) The copy number variation (CNV) of CRGs. (**D**) The location of CRGs with CNV modification on 23 chromosomes.

As shown in Fig. 2A, we observed that 7 out of 10 CRGs were differentially expressed between normal and COAD tissues, including CDKN2A, GLS, LIPT1, DLAT, DLD, FDX1, MTF1. Among them, the expression levels of CDKN2A, GLS and LIPT1 were significantly upregulated whereas the expression levels of DLAT, DLD, FDX1, MTF1 was significantly downregulated in tumor samples. In addition, we investigated genetic alterations in COAD sample. Among 374 COAD samples, 27 (7.22%) showed CRGs mutations, in which missense mutations were the dominant type (Fig. 2B). The frequencies of CRGs copy number gain and loss were presented in Fig. 2C. The CRGs sites with copy number alteration on chromosomes was visualized in Fig. 2D.

### Establishment of co-expression network and identification of CRLs

**Figure 3:**
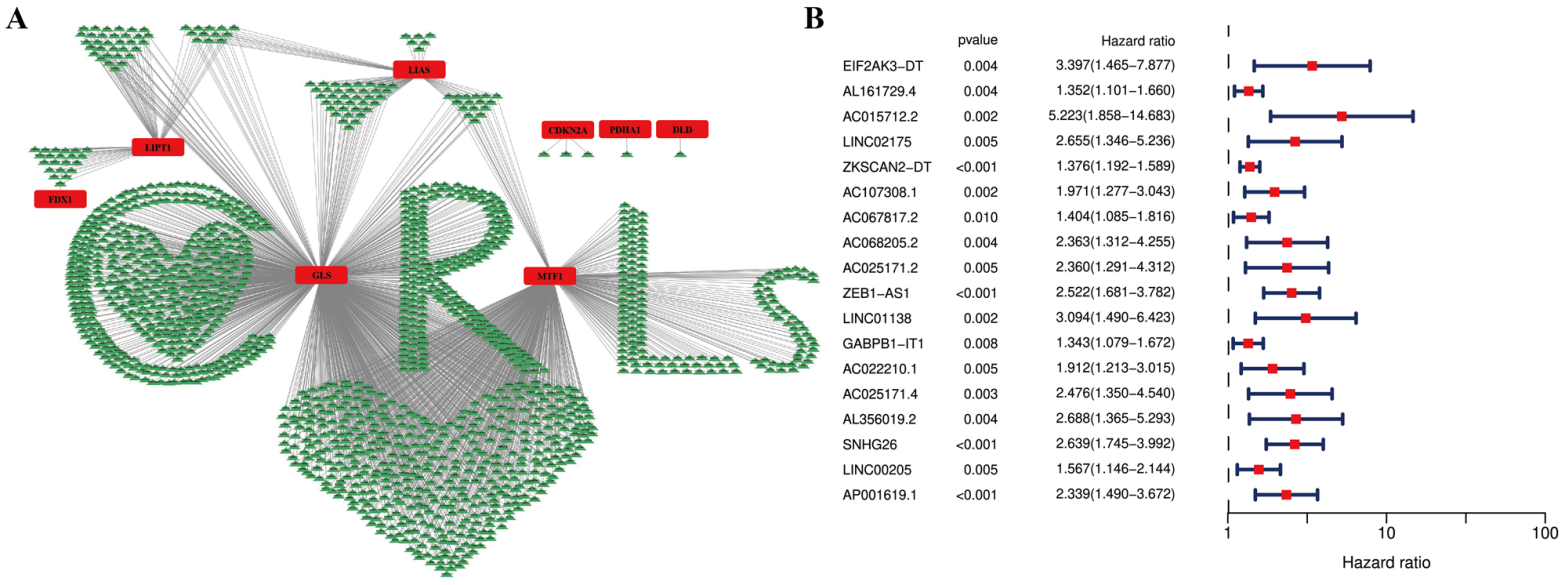
Co-expression network and prognostic cuproptosis-related lncRNAs (CRLs). (**A**) The co-expression network between cuproptosis-related genes and prognostic lncRNAs. (**B**) The forest plot of univariate Cox regression analysis for prognostic CRLs.

The transcriptomic data derived from TCGA showed that 19,790 mRNAs and 16,773 lncRNAs were identified in COAD samples. Through preforming the co-expression analysis between CRGs and lncRNAs, a total of 1410 potential CRLs were obtained (Fig. 3A, Supplementary Table S1). Subsequently, through performing Cox regression analysis, 18 CRLs correlated with prognosis of COAD patients were uncovered, including AC015712.2, AC022210.1, AC025171.2, AC025171.4, AC067817.2, AC068205.2, AC107308.1, AL161729.4, AL356019.2, AP001619.1, EIF2AK3-DT, GABPB1-IT1, LINC00205, LINC01138, LINC02175, SNHG26, ZEB1-AS1, ZKSCAN2-DT (Fig. 3B).

### Consensus clustering analysis of CRLs

**Figure 4:**
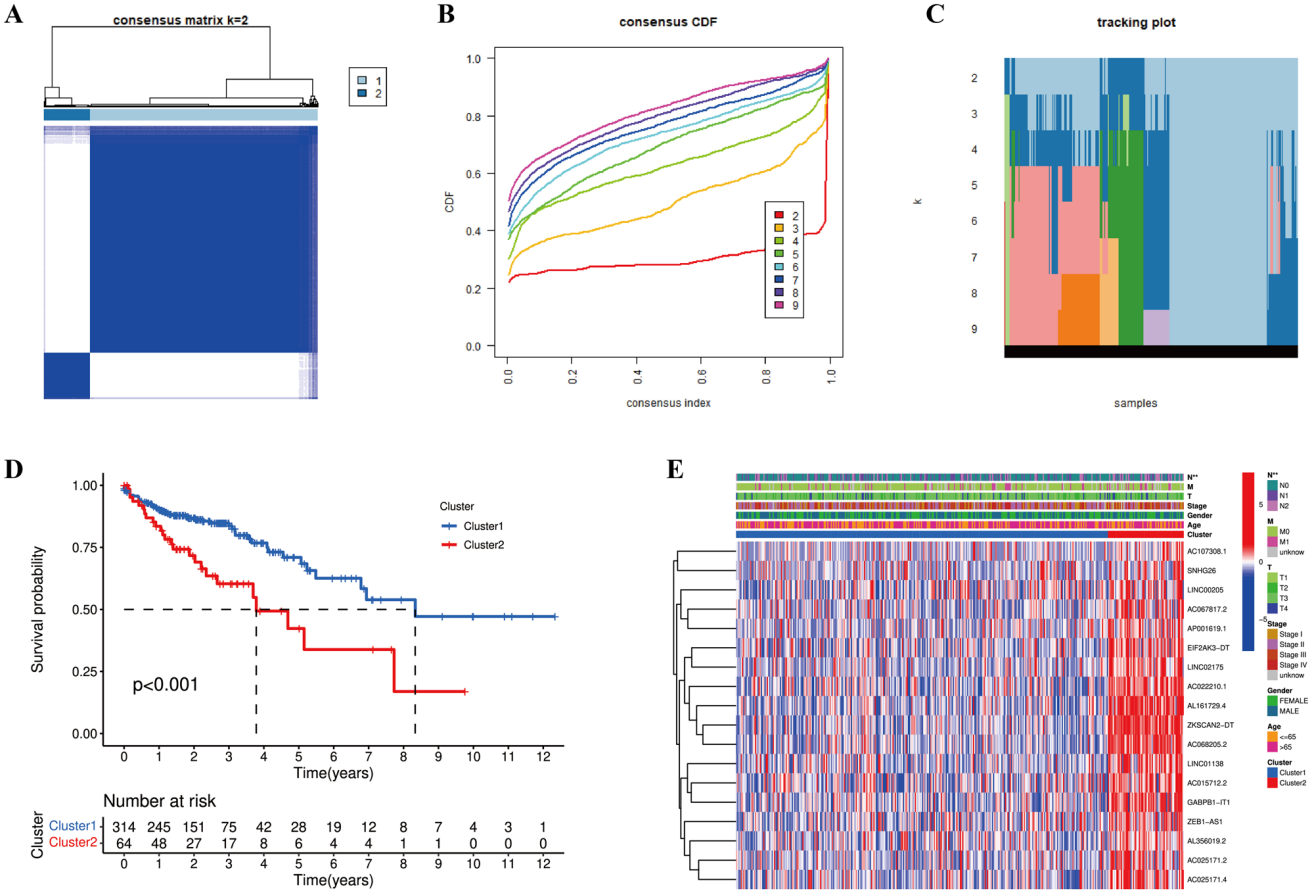
Correlation between cluster subtypes and clinical characteristics in COAD samples. (**A**–**C**) k = 2 as the optimal clustering parameter in consensus clustering analysis. (**D**) Kaplan–Meier curves of overall survival (OS) in different clusters. (**E**) Correlation analysis between cluster subtypes and clinicopathological features. **p < 0.01.

Consensus clustering analysis identified k = 2 as the optimal clustering parameter (Fig. 4A–C). Thus, all 378 patients were categorized into cluster 1 (n = 314) and cluster 2 (n = 64) (Supplementary Table S2). As presented in Fig. 4D, we found that the OS in cluster 2 was poorer than that in cluster 1 (*p* < 0.001). In addition, the correlations between different cluster subtypes and clinical features were explored based on the expression levels of CRLs. As demonstrated in heatmap, all 18 CRLs were upregulated in cluster 2, and the patients in cluster 2 had a more advanced pathological N stage than that in cluster 1 (*p* < 0.01) (Fig. 4E). Overall, these 18 CRLs could contribute to the pathological progression of COAD.

### Differences in tumor microenvironment features between the different clusters

**Figure 5:**
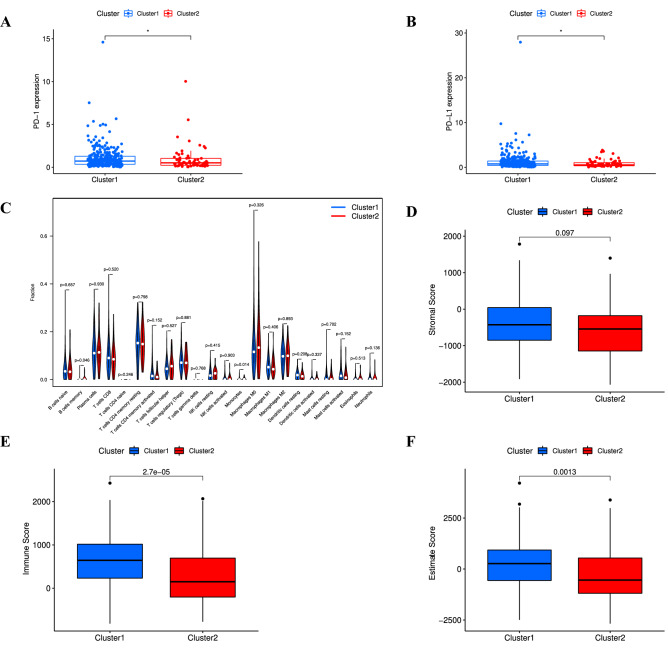
Immune analysis in different cluster subtypes. (**A**) Correlation between cluster subtypes and PD-1, *p < 0.05. (**B**) Correlation between cluster subtypes and PD-L1, *p < 0.05. (**C**) Infiltration of 22 immune cells in different cluster subtypes. (**D**–**F**) Analysis of stromal scores (**D**), immune scores (**E**), and estimated scores (**F**) in different cluster subtypes.

We observed that both PD-1 and PD-L1 expression were significantly higher in cluster 1 than that in cluster 2 (all *p* < 0.05) (Fig. 5A,B). This could mean that cluster 1 responds better to immunotherapy. Simultaneously, we revealed the differences in the abundance of 22 immune cells according to the algorithm of CIBERSORT (Supplementary Table S3). The violin diagram showed that memory B cells and monocytes were loaded with high abundance in cluster 2 (*p* < 0.05) (Fig. 5C). Though the ESTIMATE analysis demonstrated the lack of significant differences in stromal scores between the different clusters, undoubtedly, there is a trend towards the higher stromal scores in cluster 1(Fig. 5D, Supplementary Table S4). Additionally, we found cluster 1 had higher immune and estimated scores compared to cluster 2 (Fig. 5E,F), which implied lower tumor purity and more immune cell abundance in cluster 1.

### Construction of the cuproptosis-related prognostic signature

**Figure 6:**
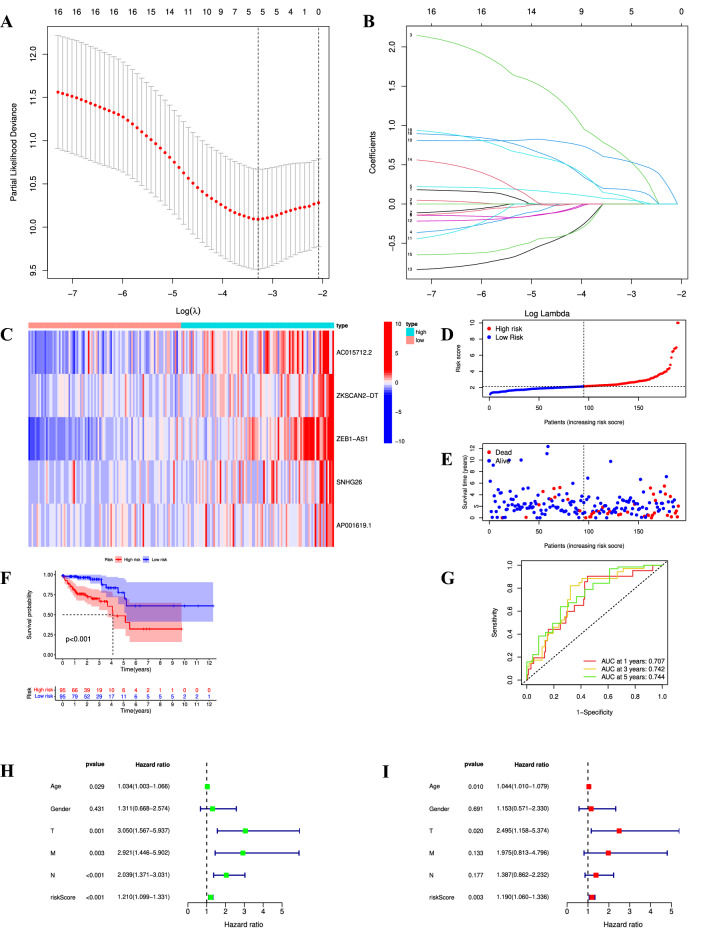
Construction of prognostic CRL signature. (**A**,**B**) Least absolute shrinkage and selection operator (LASSO) regression analysis with the most optimal lambda value. (**C**) Expression of five CRLs between high- and low-risk groups in the training cohort. (**D**,**E**) Distribution of risk score and survival states for all COAD patients in the training cohort. (**F**) Kaplan–Meier curves of OS based on the risk score in the training cohort. (**G**) Receiver operating characteristic (ROC) curve analysis in the training cohort. (**H**,**I**) Univariate Cox regression analysis (**H**) and multivariate Cox regression analysis (**I**) in the training cohort.

All 378 patients were randomly split into training cohort (n = 190) or validation cohort (n = 188). The basic clinical characteristics between two cohorts were displayed in Table 1. Through utilizing the training cohort dataset, LASSO-Cox analysis was performed to further screen prognostic CRLs and construct a CRL signature (Supplementary Table S5). Eventually, we created a five-lncRNA prognostic signature based on the optimal lambda value (Fig. 6A,B). According to the coefficient and expression level of each lncRNA, we proposed a formula to compute risk scores. Risk score = AC015712.2 × 0.678990448067854 + ZEB1-AS1 × 0.575425114920727 + SNHG26 × 0.191678723955985 + AP001619.1 × 0.119431792481994 + ZKSCAN2-DT × 0.0627395579922157.

**Table 1:** Comparison of clinical characteristics between the different cohorts.

Characteristics	Training cohort *n* (%)	Validation cohort *n* (%)	*p* value
Age			
≤ 65	82 (43.2)	74 (39.4)	> 0.05
> 65	108 (56.8)	114 (60.6)	
Gender			
Male	101 (53.2)	88 (46.8)	> 0.05
Female	89 (46.8)	100 (53.2)	
T stage			
T1	4 (2.1)	6 (3.2)	> 0.05
T2	38 (20.0)	29 (15.4)	
T3	128 (67.4)	129 (68.6)	
T4	20 (10.5)	24 (12.8)	
N stage			
N0	109 (57.4)	117 (62.2)	> 0.05
N1	41 (21.6)	46 (24.5)	
N2	40 (21.1)	25 (13.3)	
M stage			
M0	137 (72.1)	143 (76.1)	> 0.05
M1	27 (14.2)	26 (13.8)	
Mx	26 (13.7)	19 (10.1)	
TNM stage			
I	34 (17.9)	32 (17.0)	> 0.05
II	71(37.4)	78 (41.5)	
III	55 (28.9)	47 (25.0)	
IV	27 (14.2)	26 (13.8)	
Unknown	3 (1.6)	5 (2.7)

Whereafter, the patients in the training cohort were categorized into high-risk subgroup (n = 95) or low-risk subgroup (n = 95) based on the median risk score. As shown in Fig. 6C, the expression levels of all five CRLs were significantly upregulated in the high-risk subgroup. The risk scores, survival states and survival time for each patient were presented in Fig. 6D,E. As shown by K–M curves, we found that the patients with high-risk scores had poorer OS compared with the patients with low-risk scores (*p* < 0.001) (Fig. 6F). As shown by ROC curves, the 1-, 3-, and 5-year AUC values were 0.707, 0.742, and 0.744, respectively (Fig. 6G). Furthermore, univariate Cox regression analysis revealed age, T stage, N stage, M stage, and risk score were potential risk factors for COAD prognosis (all *p* < 0.05) (Fig. 6H). Multivariate Cox regression analysis revealed that age, T stage, and risk score were independent risk factors for COAD prognosis (all *p* < 0.05) (Fig. 6I).

### Verification of the cuproptosis-related prognostic signature

**Figure 7:**
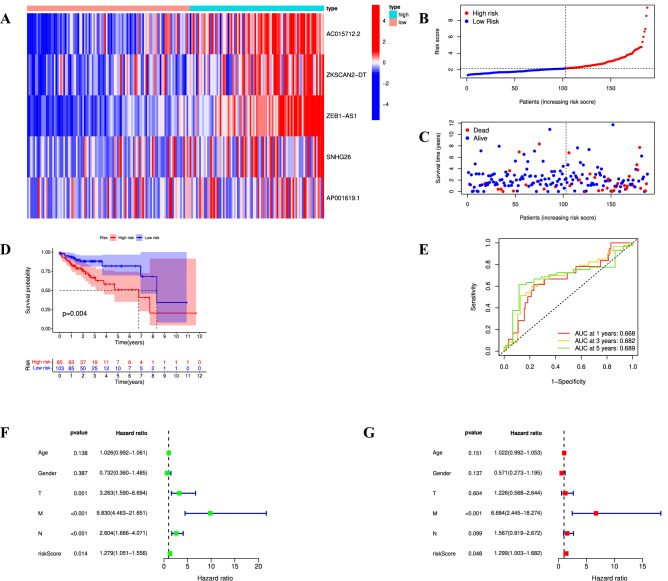
Verification of prognostic CRL signature. (**A**) Expression of five CRLs between high- and low-risk groups in the validation cohort. (**B**,**C**) Distribution of risk score and survival states for all COAD patients in the validation cohort. (**D**) Kaplan–Meier curves of OS based on the risk score in the validation cohort. (**E**) Receiver operating characteristic (ROC) curve analysis in the validation cohort. (**F**,**G**) Univariate Cox regression analysis (**F**) and multivariate Cox regression analysis (**G**) in the validation cohort.

The validation cohort data were applied to test the predictive ability of CRL signature. The patients in the validation cohort were also categorized into high-risk groups (n = 85) or low-risk groups (n = 103) by utilizing the same calculation formula and median risk score as the training cohort (Supplementary Table S6). We noticed that the expression levels of all 5 CRLs were consistent with the results of the training cohort (Fig. 7A). The risk scores, survival states and survival time for each patient in the validation cohort were also unveiled in Fig. 7B,C. Likewise, we observed poor OS in the patients with high-risk scores (Fig. 7D). The 1-, 3- and 5-year AUC values were 0.668, 0.682, and 0.689, respectively, close to 0.70 (Fig. 7E). Finally, the univariate and multivariate Cox regression analyses also confirmed that risk score was the independent risk factors for COAD prognosis (*p* < 0.05) (Fig. 7F,G).

### Construction of the nomogram to forecast the survival

**Figure 8:**
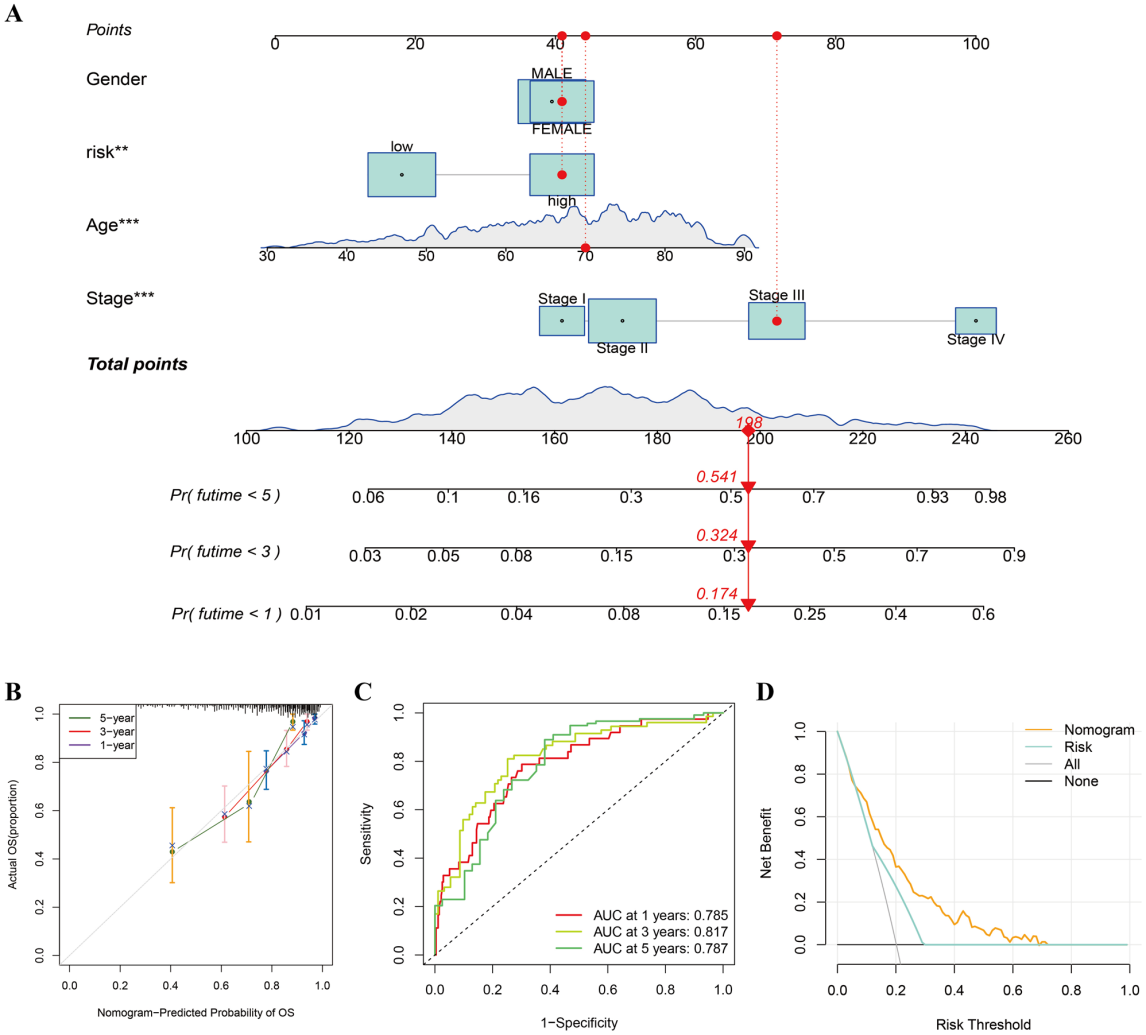
Construction of the nomogram. (**A**) Nomogram for the prediction of 1-, 3- and 5-year OS in COAD patients, **p < 0.01, and ***p < 0.001. (**B**) Calibration curves of the nomogram. (**C**) Time-dependent ROC curves of the nomogram. (**D**) Decision curve analysis of nomogram.

The nomogram was constructed to forecast the 1-, 3- and 5-year OS of COAD patients based on the clinical information, including age, gender, TNM stage, and signature-calculated risk scores. The survival probabilities in different periods were clearly shown in Fig. 8A. For example, for a 70-year-old female with tumor stage III categorized as high-risk based on our CRL signature, the OS probabilities less than 1, 3, and 5 years were 0.174, 0.324, and 0.541, respectively. The results of 1-, 3-, and 5-year calibration curves demonstrated the nomogram-predicted OS was well matched with the best prediction performance (Fig. 8B). The time-dependent ROC curves of nomogram demonstrated the 1-, 3- and 5-year AUC values were 0.785, 0.817, and 0.787, respectively (Fig. 8C). These findings suggest the prognostic nomogram has a significant predictive value for 1, 3, and 5-year survival in patients with COAD. Additionally, DCA analysis showed the nomogram harbored significantly the best clinical net benefit when the threshold probability is greater than 0.1 (Fig. 8D).

### Correlation between risk signature and clinical characteristics

**Figure 9:**
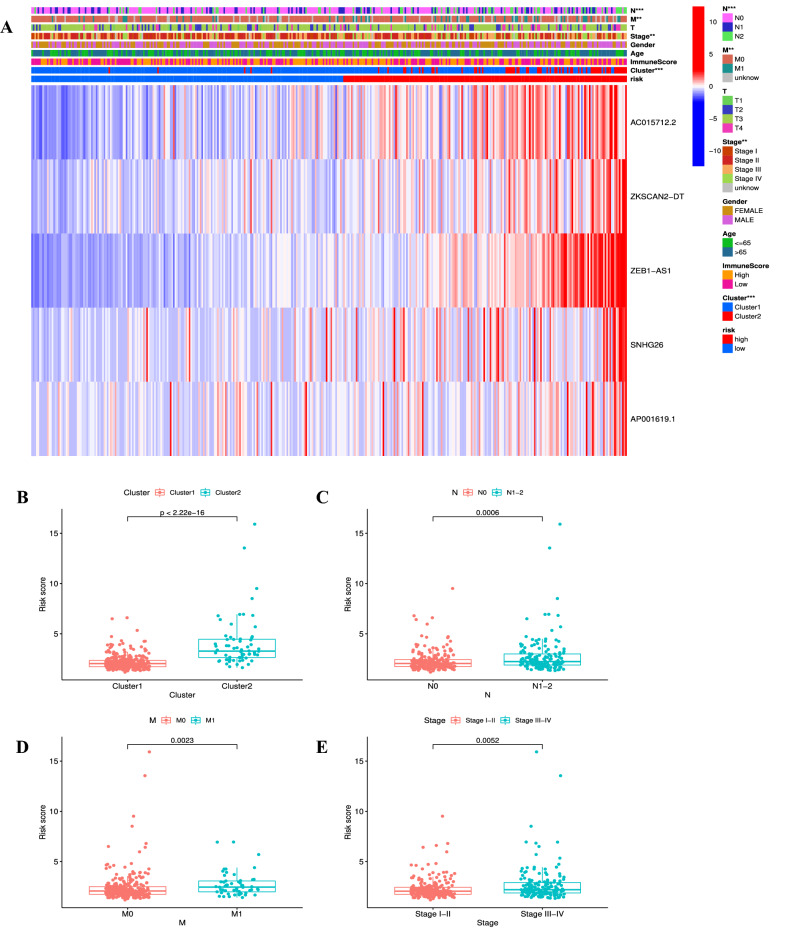
Correlation between prognostic signature and clinical characteristics. (**A**) The heatmap for the distribution clinical characteristics in different risk groups. (**B**–**E**) The histogram for distribution of risk score with significant difference stratified by cluster (**B**), pathological N stage (**C**), pathological M stage (**D**), and tumor stage (**E**). **p < 0.01, and ***p < 0.001.

As described in the heatmap, the strong correlations between risk scores and patients’ clinical characteristics were confirmed (Fig. 9A). Specifically, we discovered that high risk scores were significantly correlated with cluster 2 (Fig. 9B), N1–2 stage (Fig. 9C), M1 stage (Fig. 9D), and TNM III–IV stage (Fig. 9E) (all *p* < 0.05). These findings further illustrated that CRLs used to construct this prognostic signature could promote tumor progression and metastasis in COAD patients.

### Pathway enrichment analysis

**Figure 10:**
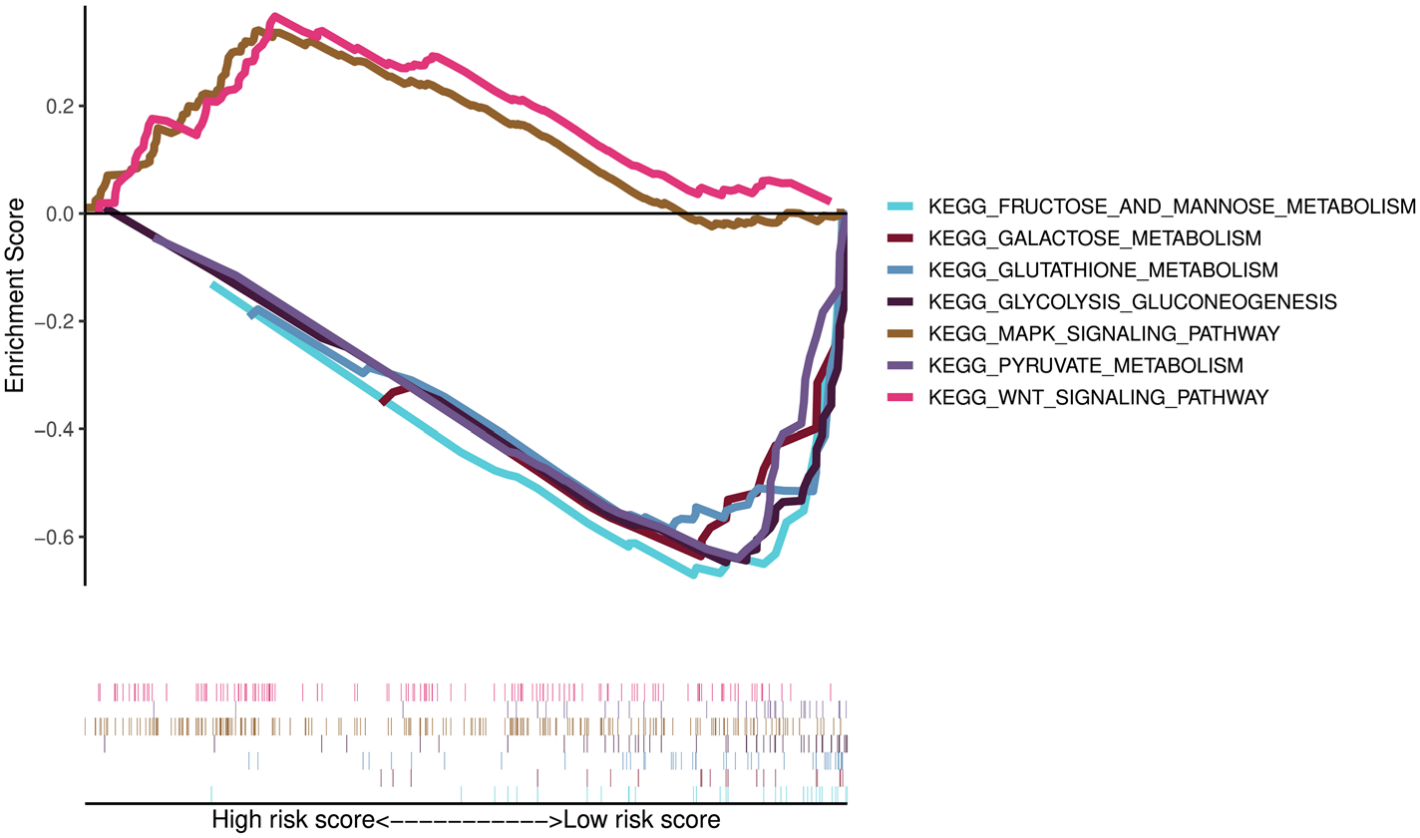
Gene set enrichment analysis (GSEA) based on KEGG pathway in different risk groups.

We performed GSEA to seek whether there were differences in signaling pathways between different risk subgroups. As illuminated in Fig. 10, GSEA unveiled two KEGG pathways, including “MAPK signaling pathway” and “Wnt signaling pathway”, were significantly enriched on the high-risk score side. Conversely, on the other side with low-risk score, many metabolic pathways were significantly concentrated, with the top five enriched pathways including “galactose metabolism”, “glutathione metabolism”, “pyruvate metabolism”, “fructose and mannose metabolism”, and “glycolysis gluconeogenesis” (Supplementary Table S7).

### Immune analysis of risk signature

**Figure 11:**
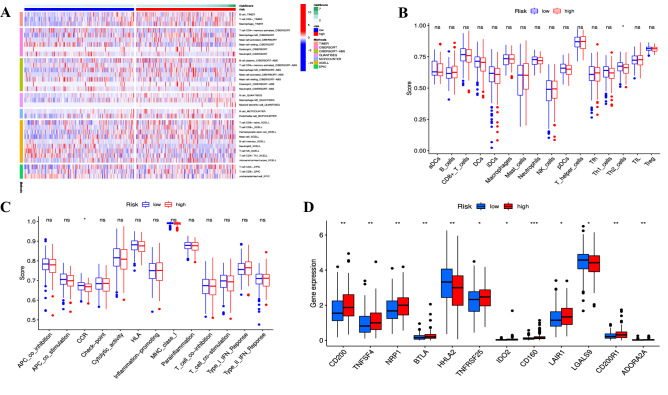
Immune correlation analysis based on CRL signature between high- and low-risk groups. (**A**) Analysis of immune cell infiltration with significant difference in different risk groups. (**B**) The differences in the scores of immune cells in different risk groups. (**C**) The differences in the scores of immune functions in different risk groups. (**D**) The differential expression of immune checkpoints in different risk groups. *p < 0.05, **p < 0.01, and ***p < 0.001.

The infiltration of immune cells with statistical significance was demonstrated in the heatmap (Fig. 11A, Supplementary Table S8). Multiple immune cells infiltrated more in the high-risk subgroup, such as CD4^+^ T cells, CD8^+^ T cells, B cells, mast cells, and M2 macrophages, whereas neutrophils infiltrated more in the low-risk subgroup (all *p* < 0.05). Moreover, the scores of immune cells and immune functions indicated that Th2 cells and chemokine receptors (CCR) were more abundant in the low-risk subgroup (all *p* < 0.05) (Fig. 11B,C). As for the expression levels of immune checkpoint genes, except for HHLA2 and LGALS9, which were downregulated in the high-risk subgroup, many immune checkpoints were upregulated in the high-risk subgroup, including CD200, TNFSF4, NRP1, BTLA, TNFRSF25, IDO2, CD160, LAIR1, CD200R1, ADORA2A (all *p* < 0.05) (Fig. 11D). Hence, targeted cuproptosis combination with immunotherapy may provide a novel strategy for COAD treatment in the future.

### Methylation correlation analysis of risk signature

**Figure 12:**
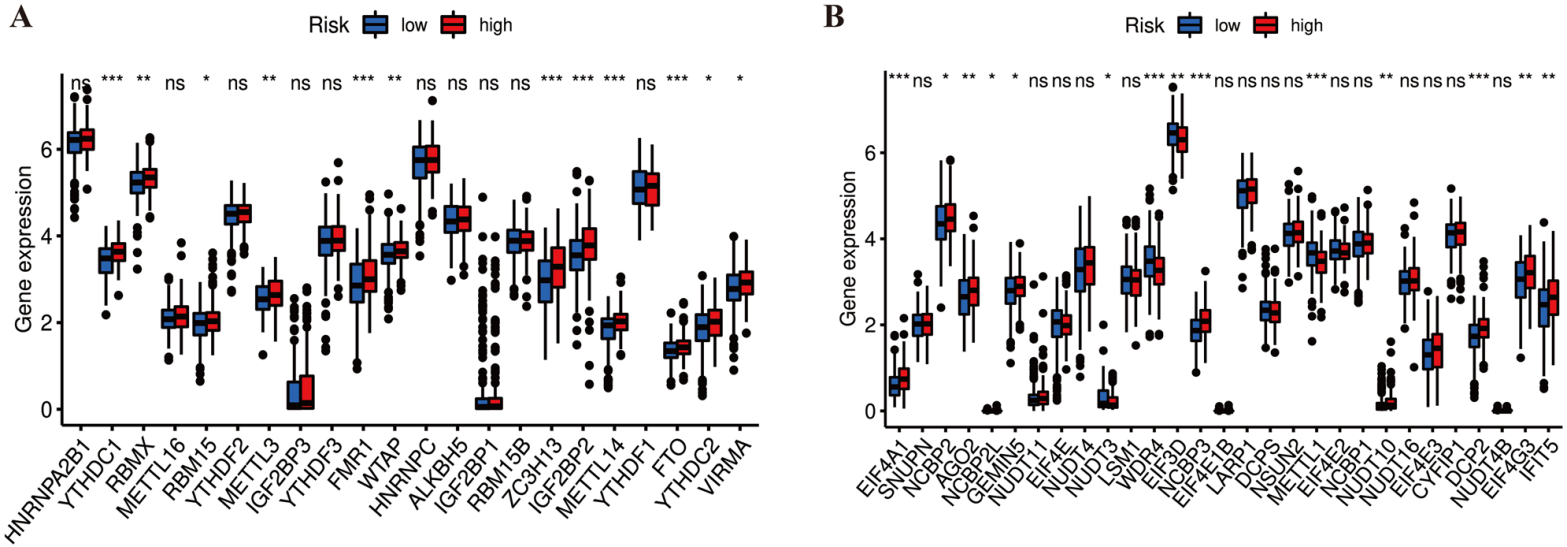
Methylation correlation analysis of *N*6-methyladenosine (m6A) and *N*7-methylguanosine (m7G) in high- and low-risk groups. (**A**) Differential expression of m6A-related genes in different risk groups. (**B**) Differential expression of m7G-related genes in different risk groups. *p < 0.05, **p < 0.01, and ***p < 0.001.

The correlation between risk signature and RNA methylated modifications were analyzed using m6A- and m7G-related gene sets derived from previous literature^32,33^. As shown in Fig. 12A, 12 out of 22 m6A-related genes were upregulated in the high-risk subgroup, including FMR1, FTO, IGF2BP2, METTL3, METTL14, RBM15, RBMX, VIRMA, WTAP, YTHDC1, YTHDC2, ZC3H13 (all *p* < 0.05). As shown in Fig. 12B, half of the m7G-related genes were significantly differentially expressed, most of which were upregulated in the high-risk group, including AGO2, DCP2, EIF4A1, EIF4G3, GEMIN5, IFIT5, NCBP2, NCBP2L, NCBP3, NUDT10 (all *p* < 0.05).

### Drug sensitivity analysis

**Figure 13:**
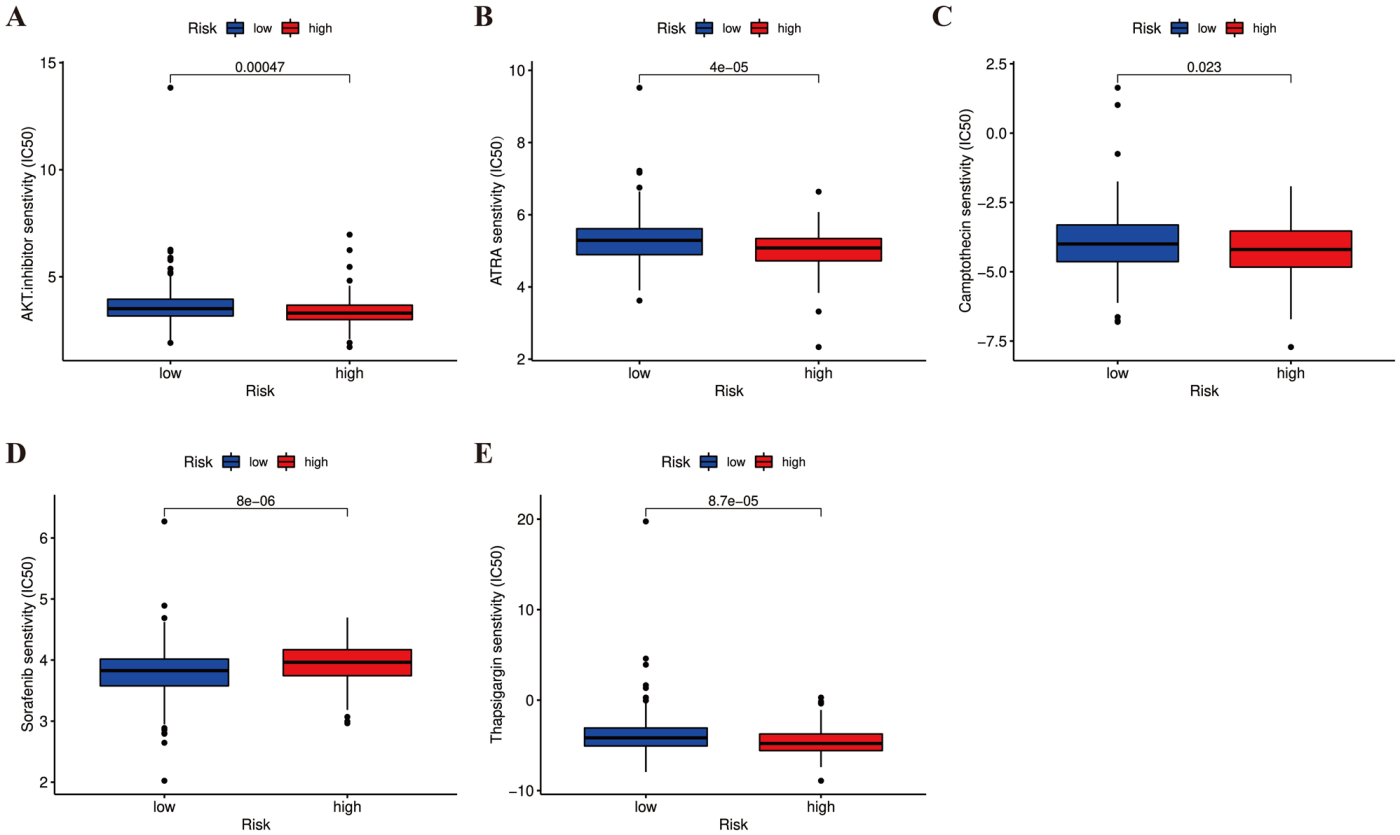
Drug sensitivity and potential therapeutic agents in high- and low-risk groups. AKT inhibitors (**A**), all-trans retinoic acid (**B**), camptothecin (**C**), sorafenib (**D**), and thapsigargin (**E**).

We screened for sensitivity to a range of antitumor drugs, involved in chemotherapeutics, targeted agents, and small molecule drugs. As illuminated in Fig. 13, except for sorafenib that was more sensitive in the low-risk group, we found the AKT inhibitors, ATRA, camptothecin, and thapsigargin were relatively more sensitive in the high-risk group (all *p* < 0.05). These findings of can provide reference for clinical treatment of COAD.

## Discussion

Colon cancer, an infamous malignancy originating from the colonic epithelial cells, is one of the primary causes of tumor-related death in globally^34^. Though individualized treatment for colon cancer has been recognized, the classification criteria, prognosis assessment, and treatment plan of colon cancer still mainly depend on histopathological characteristics in clinical practice^35^. Therefore, it is imperative to seek practical molecular biomarkers. Recently, cuproptosis has been identified as a novel modality to trigger cell death by mitochondrial lipoylated proteins^7^. However, the current understanding of the association between cuproptosis and tumorigenesis and progression remains limited.

In this study, based on transcript dataset derived from the TCGA portal, we screened the prognostic CRLs in colon adenocarcinoma. In the co-expression network, we observed that GLS, a negatively regulatory gene of cuproptosis, had a strong correlation with most lncRNAs, which meant GLS could play the role of hub gene for these essential CRLs. GLS encodes glutaminase that catalyzes the hydrolysis of glutamine to glutamate to supplement TCA cycle intermediates. Accumulating evidence confirmed that glutaminase was closely related to tumor cell growth and proliferation^36,37^. A recent study elucidated that GLS was overexpressed in multiple tumor cells (including colon cancer), which were linked to unfavorable prognosis and could serve as a tumor biomarker^38^. Consistent with previous studies, our work confirmed that GLS expression was upregulated in colon tumor tissues, and GLS copy number gain was greater than copy number loss.

Furthermore, we established a novel CRL signature for foreseeing prognosis of COAD via LASSO-Cox regression analysis. We witnessed the superior efficacy and accuracy of this CRL signature in foreseeing the prognostic by validating the risk model. Specially, we noticed that all five CRLs were upregulated expressed in COAD samples and risk score emerged as an independent risk factor for prognosis. This meant that five CRLs could be prognostic biomarkers and potential targets for drugs in the patients with COAD.

We noticed that 3 CRLs, ZEB1-AS1, SNHG26, and AP001619.1, which were used to construct this prognostic signature, were previously reported to be related to tumors. (1) Li et al. concluded that, ZEB1‐AS1 as an oncogenic regulator, not only upregulated ZEB1 expression to induce epithelial-mesenchymal transition (EMT), but also activated IL-11/STAT3 signaling to cause uncontrolled proliferation, metastasis, and anti-apoptosis of tumor cells^39^. A meta-analysis indicated that the high expression of ZEB1-AS1, as an adverse factor of cancer prognosis, was significantly positively associated with worse differentiation, deeper invasion, earlier metastasis, poorer clinical stage, and shorter survival^40^. (2) Jiang et al. revealed that SNHG26 stimulated PGK1/AKT/mTOR signaling pathway to promote tumor growth, metastasis, and chemotherapy resistance^41^. Hegre et al. illuminated that knockdown of SNHG26 could interfere with the phase distributions of the cell cycle and reduce cell proliferation^42^. It is worth noting that a recent study analogously used SNHG26 to establish a prognostic signature of tumor immune infiltration-related lncRNAs in colon cancer, which confirmed the indispensable role of SNHG26 in the prognosis^43^. (3) Previous studies on colon cancer have demonstrated a significant association between AP001619.1 and prognosis by creating a competing endogenous RNAs network and multi-RNA-based signature^44^. Xu et al. screened 10 CRLs to foresee the prognosis of colon cancer, among which AP001619.1 was included in the prognostic model^45^. Forementioned studies provide evidence to support the components of our prognostic model. For the other two CRLs, including AC015712.2 and ZKSCAN2-DT, it’s the first time to report their prognostic roles in cancer. Hence, how these CRLs affect the prognosis remains to be further studied.

The GSEA revealed that MAPK and Wnt signaling pathways were significantly positively correlated with high-risk scores. This means our findings may be beneficial for shedding light on the underlying tumorigenic mechanism of CRLs. Studies reported that copper can bind MEK1/2 and enhance the phosphorylation of MEK1/2 in a dose-dependent manner, thereby affecting the strength of the oncogenic RAF-MEK-ERK cascade^46,47^. Lv et al. uncovered that lncRNA ZEB1-AS1, a competing endogenous RNA of miR-181a-5p, promoted colorectal tumor cells proliferation and anti-apoptosis by regulating Wnt/β-catenin signaling^48^. These potential mechanisms can provide reference for tumor targeted therapy.

Tumor cells can suppress antitumor immune responses by activating the immune checkpoint pathway and thus escape immunological surveillance^49^. Growing evidence reportes that immune checkpoint inhibitors provide durable clinical benefits in comprehensive therapy of colon cancer^50,51^. In this study, we observed many immune checkpoints were upregulated in the high-risk subgroup, suggesting that the regulatory role of CRLs in immunotherapy remains to be further investigated. We also noticed that the patients in high-risk subgroup had more infiltration of multiple immune cells, such as CD4^+^ T cells, CD8^+^ T cells, B cells, mast cells, and M2 macrophages. In addition, ssGSEA indicated that the abundance of Th2 cells and CCR was lower in the high-risk subgroup. Overall, we speculated a more complex tumor microenvironment might be shaped in the high-risk group. These results suggested the association between cuproptosis and tumor immunity.

Evidence demonstrates that RNA methylation is closely related to cell proliferation, metastasis, and immune response^52^. Thus, we investigated the correlation between risk signature and m6A- and m7G-related genes. In the high-risk group, we noticed many m6A- and m7G-related genes were upregulated, which might lead to dysregulation of methylation and affect the survival of COAD patients. Our results help to build a bridge and reveal the potential association between cuproptosis and RNA methylation.

To find suitable drugs to meet the needs of individualized therapy and improve the prognosis of COAD patients, we screened a series of chemotherapy and targeted drugs based on different risk characteristics. The results indicated that AKT inhibitors, ATRA, camptothecin, and thapsigargin were more sensitive in high-risk patients. In contrast, sorafenib was more sensitive in the low-risk patients. Hence, AKT inhibitors, ATRA, camptothecin, and thapsigargin are promising as the effective antitumor treatment for patients with high expression of CRLs. It is well known that the abnormal activation of the PI3K/AKT/mTOR signaling pathway closely related to the tumorigenesis and progression. Evidence has suggested the oncogenic effects of ZEB1-AS1 are attributable to activation of the PI3K/AKT/mTOR pathway^53^. Downregulating ZEB1-AS1 can markedly suppress cell proliferation, invasion, and migration through inhibiting the PI3K/AKT/mTOR via miR-342-3p/Cullin 4B Axis^54^. Furthermore, ZEB1-AS1 activates the PI3K/AKT signaling by targeting miR-302b, leading to increased expression of downstream matrix metalloproteinase, which is closely relevant to tumor invasion and metastasis^55^. Regarding SNHG26, it promotes cisplatin resistance in tumor cells by activating the AKT/mTOR signaling pathway^41^. These may be the reasons why patients with high 5-CRL risk scores are sensitive to AKT inhibitors. Nevertheless, the roles and mechanisms of the 5 CRLs in other sensitive drugs remains unclear, which remains to be further investigated in the future.

This study has the following limitations. First, since the data utilized to establish the risk model were derived from a single TCGA database, this study lackd external clinical samples to verify the results. Second, basic experiments should be performed to further reveal the underlying molecular mechanisms for how these lncRNAs affect cuproptosis.

## Conclusions

In conclusion, we established a CRL signature with five CRLs to forecast the prognosis of colon adenocarcinoma. We preliminarily identified the association between this CRL signature and the tumor microenvironment. The findings provide valuable insights into the survival prediction and offer novel strategies for mechanistic exploration of cuproptosis. It may even hold the promise of improving therapeutic approaches for colon cancer patients in clinical practice.

## Supplementary Information

Supplementary Table S1.

Supplementary Table S2.

Supplementary Table S3.

Supplementary Table S4.

Supplementary Table S5.

Supplementary Table S6.

Supplementary Table S7.

Supplementary Table S8.

## Data Availability

The original data for this study were obtained from TCGA database (https://portal.gdc.cancer.gov). All data generated or analysed during this study are included in this published article and its supplementary information files. Further inquiries can be directed to the corresponding authors.
